# The importance of adjusting for enterococcus species when assessing the burden of vancomycin resistance: a cohort study including over 1000 cases of enterococcal bloodstream infections

**DOI:** 10.1186/s13756-018-0419-9

**Published:** 2018-11-14

**Authors:** Tobias Siegfried Kramer, Cornelius Remschmidt, Sven Werner, Michael Behnke, Frank Schwab, Guido Werner, Petra Gastmeier, Rasmus Leistner

**Affiliations:** 1 Charité Universitätsmedizin Berlin, Institute of Hygiene and Environmental Medicine, Berlin, Germany; 2 National Reference Center for the Surveillance of Nosocomial Infections, Berlin, Germany; 3 Department of Medical and Financial Controlling, Charité Universitätsmedizin Berlin, Berlin, Germany; 4 Robert Koch Institute, FG13 Nosocomial Pathogens and Antibiotic Resistance, Wernigerode, Germany; 5 National Reference Centre for Staphylococci and Enterococci, Berlin, Germany

**Keywords:** Bloodstream infection, Vancomycin-resistant enterococci, *Enterococcus faecium*

## Abstract

**Background:**

Infections caused by vancomycin-resistant enterococci (VRE) are on the rise worldwide. Few studies have tried to estimate the mortality burden as well as the financial burden of those infections and found that VRE are associated with increased mortality and higher hospital costs. However, it is unclear whether these worse outcomes are attributable to vancomycin resistance only or whether the enterococcal species (*Enterococcus faecium* or *Enterococcus faecalis*) play an important role. We therefore aimed to determine the burden of enterococci infections attributable to vancomycin resistance and pathogen species (*E. faecium* and *E. faecalis)* in cases of bloodstream infection (BSI).

**Methods:**

We conducted a retrospective cohort study on patients with BSI caused by *Enterococcus faecium* or *Enterococcus faecalis* between 2008 and 2015 in three tertiary care hospitals. Data was collected on true hospital costs (in €), length of stay (LOS), basic demographic parameters, and underlying diseases including the results of the Charlson comorbidity index (CCI). We used univariate and multivariable regression analyses to compare risk factors for in-hospital mortality and length of stay (i) between vancomycin-susceptible *E. faecium-* (VSEm) and vancomycin-susceptible *E. faecalis-* (VSEf) cases and (ii) between vancomycin-susceptible *E. faecium-* (VSEm) *and* vancomycin-resistant *E. faecium-cases* (VREm). We calculated total hospital costs for VSEm, VSEf and VREm.

**Results:**

Overall, we identified 1160 consecutive cases of BSI caused by enterococci: 596 (51.4%) cases of *E. faecium* BSI and 564 (48.6%) cases of *E. faecalis* BSI. 103 cases of *E. faecium* BSI (17.3%) and 1 case of *E. faecalis* BSI (0.2%) were infected by vancomycin-resistant isolates. Multivariable analyses revealed (i) that in addition to different underlying diseases *E. faecium* was an independent risk factor for in-hospital mortality and prolonged hospital stay and (ii) that vancomycin-resistance did not further increase the risk for the described outcomes among *E. faecium*-isolates. However, the overall hospital costs were significantly higher in VREm-BSI cases as compared to VSEm- and VSEf-BSI cases (80,465€ vs. 51,365€ vs. 31,122€ *p* < 0.001).

**Conclusion:**

Our data indicates that in-hospital mortality and infection-attributed hospital stay in enterococci BSI might rather be influenced by Enterococcus species and underlying diseases than by vancomycin resistance. Therefore, future studies should consider adjusting for Enterococcus species in addition to vancomycin resistance in order to provide a conservative estimate for the burden of VRE infections.

**Electronic supplementary material:**

The online version of this article (10.1186/s13756-018-0419-9) contains supplementary material, which is available to authorized users.

## Introduction

Enterococcus spp. are part of the normal gastrointestinal flora. Among those pathogens, resistance to antimicrobial substances, notably to vancomycin, results in limited therapeutic options [1, 2]. In recent years, hospital-acquired infections (HAI) caused by vancomycin-resistant enterococci (VRE) have emerged as a relevant burden on patients and healthcare systems globally [3–5]. In order to reduce the spread of resistant strains in hospitals, infection control measures, e.g. contact precautions, have been proposed [6, 7]. To assess the efficiency of VRE prevention measures, the mortality- and financial burden of VRE infections has to be assessed. However, the methodological approach on assessing VRE-burden remains controversial [2, 8, 9] and only few studies have addressed economic aspects [10–15]. As costs are often not available as infection-attributable costs (costs after onset for infection) length of stay (LOS) after onset of infection is being used as a surrogate parameter [2, 8, 16].

Although analyses should compare VRE infections to VSE-infected patients when the attributable effect of vancomycin resistance is addressed, [2, 8, 16] prior studies also utilized comparisons to cohorts with non-enterococcus infections [17, 18] or cohorts without infection [8, 12, 19–24]. Since the course of enterococcal infections may also be influenced by the enterococcus subspecies itself [2, 8, 9, 25, 26], analyses not considering the pathogen species may therefore be biased as result of the different virulence of the pathogens.

In a large cohort of cases with bloodstream infection (BSI), we therefore studied the influence of vancomycin resistance and enterococcus subspecies on in-hospital mortality, hospital costs and length of hospital stay.

## Methods

### Setting, study design and data collection

The study was conducted at three different tertiary care hospitals of the Charité university hospital in Berlin, with 3011-beds in total [27]. After a confirmatory ethics vote was obtained from the Charité University Medicine ethics committee (internal processing key EA4/229/17), we performed a cohort study that included all cases of BSI caused by *Enterococcus faecalis* or *Enterococcus faecium* between January 1, 2008 and December 31, 2015. Cases were identified in the Charité microbiology database as hospitalized patients with blood cultures positive for one of these pathogens. Data on costs and hospital financial accounting was provided by the Charité Department of Financial Controlling as true hospital expenses in Euros. For all patients enrolled in this study, the following demographic and clinical characteristics were collected: age, sex, in-hospital death, length of hospital stay (LOS), day of BSI onset and stay on an intensive care unit (days). Length of stay in total and after BSI onset were defined as length of stay until death or discharge. The Charlson comorbidity index (CCI) was obtained on the basis of the patients’ diagnosed comorbidities using the method of Charlson et al. and the adaptation for the ICD-10 by Thygesen et al. [28, 29]. The original 17 Charlson comorbidity categories were cumulated based on the affected organ system in the following ten disease categories: heart disease, cerebrovascular disease, neurologic disease, lung disease, rheumatic disease, gastrointestinal disease, liver disease, diabetes, renal disease and cancer/immunological disease.

### Definitions and statistical methods

Cases were defined as patients with BSI caused by Enterococcus spp. *(Enterococcus faecalis* or *Enterococcus faecium)* during the study period. Each patient was included in the analysis once. Onset of BSI was defined as the date of the first blood culture positive for the respective pathogen. BSI was considered hospital-onset if it occurred after the third day of hospitalization. Mortality was assessed based on discharge alive or in-hospital death. Data on hospital costs were derived from true hospital costs (hospital expenses). The costs analyzed cover direct costs to the hospital of treatment and diagnostics as well as indirect hospital costs of activities without patient contact (e.g. administration, hospital maintenance). The estimated cost of individual cases was based on definite performances and on settlement keys (e.g. nurse working time per patient). Economic data was available on total hospital costs and on daily costs. A differentiation of costs before and after the infection was not available. However, as length of stay directly correlates with hospital costs, length of stay after onset for infection can be applied as proxy for infection attributable additional expenditures [2, 30, 31]. We therefore assessed the multiplicative effect on length of stay after BSI onset in a multivariable linear regression.

Descriptive, univariate analyses were performed for the total cohort, stratified by enterococcus species (i.e. vancomycin susceptible *E. faecium* vs. vancomycin susceptible *E. faecalis*) and by vancomycin susceptibility (i.e. vancomycin susceptible *enterococci* vs. vancomycin resistant enterococci). Since among E. faecalis-isolates only one isolate was resistant against vancomycin, only *E. faecium* (VSEm vs. VREm) were analyzed in the second analysis. Additionally, we compared in univariate analysis deceased patients and patients discharged alive. The median and the interquartile range (IQR) were calculated for continuous parameters; number and percentage were calculated for binary parameters. Univariate differences were tested using the Wilcoxon rank-sum test for continuous variables and the Chi-square test for binary variables.

The two linear regression analyses were performed for length of stay (LOS) after onset of enterococcal BSI by stepwise forward variable selection. The continuous parameters LOS after onset of BSI was log transformed to achieve normal distribution. Only surviving patients were included. Parameters considered in the full model were vancomycin resistance OR pathogen species (*E. faecium* or *E. faecalis*), sex, age and all underlying diseases assessed as described above. From the full model, parameters with the smallest Chi-square statistic and *p* > 0.05 in the type III test were removed. The regression coefficients were converted to the measures of effect using an exponential transformation and referred to as the multiplicative effect (ME) of investigated parameters. *P*-values < 0.05 were considered significant.

Multivariable, binary logistic regression was performed for in-hospital death by stepwise forward selection. The *p*-values for including a variable in the model was 0.05 and for excluding 0.06 respectively. Odds ratios (HR) with 95% confidence intervals (95% CI) were calculated. Parameters considered in the model were sex, age, pathogen species (*E. faecium* vs. *E. faecalis*) with the interaction of the vancomycin resistance and all underlying diseases assessed as described above.

All analyses were performed using SPSS (IBM SPSS statistics, Somer, NY, USA) and SAS (SAS Institute, Cary, NC, USA).

### Microbiological methods

If a blood stream infection was suspected, blood cultures were drawn and incubated for up to seven days using standard blood culture tubes (BACTEC, Becton Dickinson Heidelberg Germany). If growth was detected, gram staining and culturing were performed. MALDI TOF MS and Vitek 2 automated system (Biomerieux Marcy l’etoile France) were used for identification and susceptibility testing of bacterial strains. They were interpreted using EUCAST definitions.

## Results

**Fig. 1:**
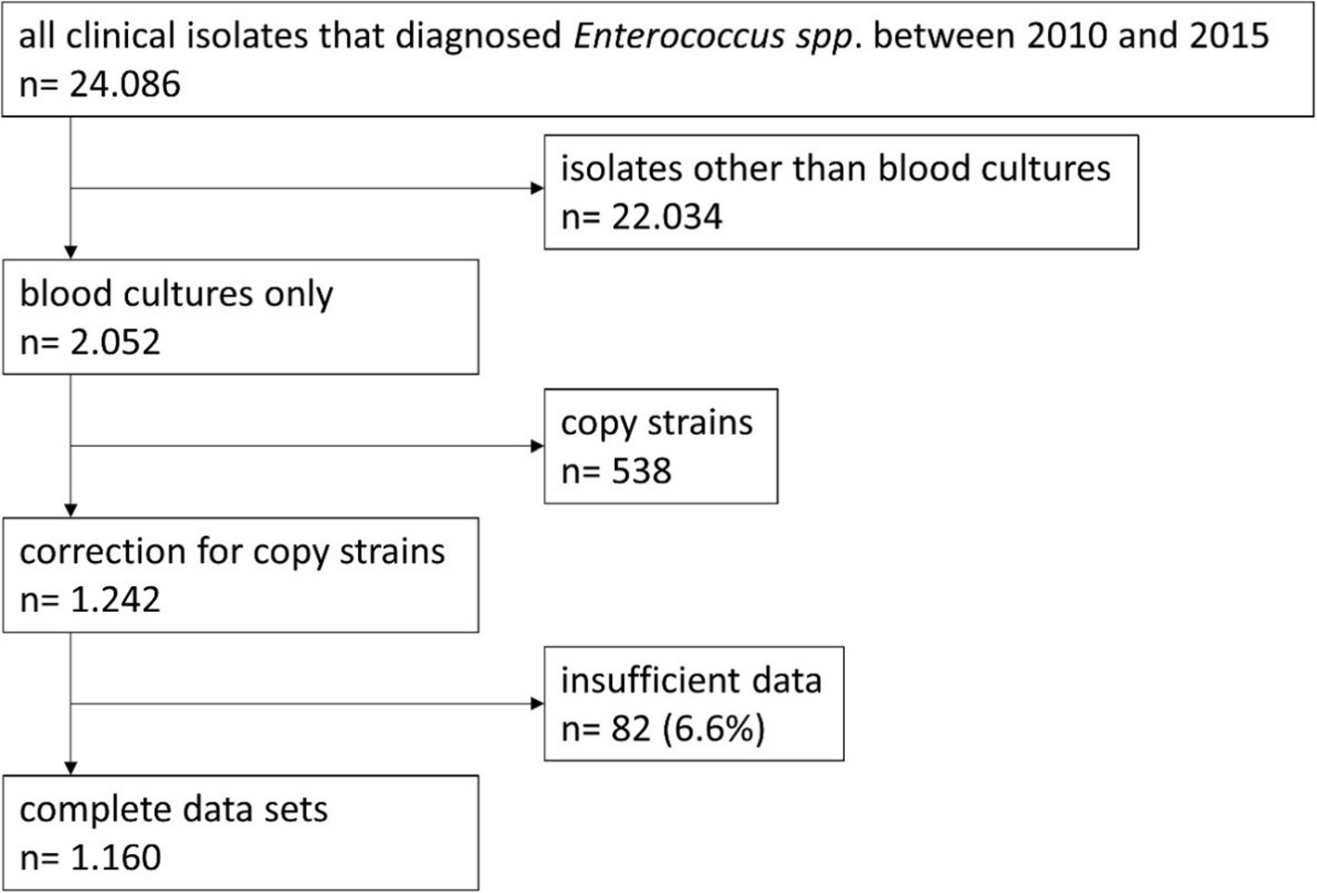
Flowchart depicting patient recruitment based on blood culture isolates

We initially extracted *n* = 24,086 clinical isolates diagnosed with *Enterococcus faecium or Enterococcus faecalis* from the microbiology database. After excluding all isolates not derived from blood cultures and correcting for copy strains, 1242 patients with BSI caused by *E. faecium* or *E. faecalis* were included in the analysis (Fig. 1). Sufficient data on all relevant parameters was available for 96.4% of the patients. Overall, this accounted for 1160 patients, 91% with infections caused by VSE and 9% by VRE. Table 1 gives an overview of the parameters for all patients and shows the results of the (i) univariate comparison of VSEm vs. VSEf and (ii) the comparison of VSEm vs. VREm BSI cases. The highest in-hospital mortality rate was found among VREm cases (50.5%) followed by VSEm cases (39.6%) and VSEf cases (24.4%). Also regarding LOS, highest numbers were found among VREm cases (total LOS, 54 days) followed by VSEm (42 days) and VSEf (32 days). In all three groups, the Charlson comorbidity score was similar with a median of 7.

**Table 1:** Univariate analysis of epidemiologic parameters, length of stay, and direct hospital costs of cases with blood stream infection caused by enterococcus spp. stratified by vancomycin resistance

Parameter	(A) VS-*E. faecium* (*n* = 493)	(B) VS-*E. faecalis* (*n* = 563)	*P-Value for A)* vs. *B)*		(C) VR-*E. faecium (n = 103)*	*P-Value for C)* vs. *A)*	
			*P-Value*	*OR (CI 95)*		*P-Value*	*OR (CI 95)*
In-hospital mortality	39.6% (195)	24.4% (132)	**0.000**	**2.137 (1.638-2.787)**	50.5% (52)	**0.041**	**1.558 (1.017–2.387)**
Male	58.6% (289)	63.1% (355)	0.141	0.830 (0.648-1.063)	66.0% (68)	0.163	1.371 (0.879–2.141)
Age in years, median (IQR)	67 (54–75)	64 (53–73)	0.059	n.a.	61 (52–70)	0.107	
Cardiac disease	28.2% (139)	29.0% (163)	0.786	0.964 (0.737-1.259)	26.2% (27)	0.683	0.905 (0.559-1.464)
Vascular disease	22.1% (109)	22.4% (126)	0.916	0.984 (0.736-1.317)	22.3% (23)	0.961	1.013 (0.608-1.687)
Pulmonary disease	20.1% (99)	23.1% (130)	0.236	0.837 (0.623-1.124)	21.4% (22)	0.769	1.081 (0.643-1.818)
Rheumatic disease	3.0% (15)	3.6% (20)	0.644	0.852 (0.431-1.683)	2.9% (3)	0.944	0.956 (0.272-3.364)
Gastrointestinal disease	5.5% (27)	3.7% (21)	0.174	1.495 (0.834-2.680)	5.8% (6)	0.888	1.068 (0.429-2.656)
Diabetes	22.9% (113)	32.0% (180)	**0.001**	**0,633 (0,481-0,833)**	19.4% (20)	0.437	0,810 (0.476-1.379)
Renal disease	59.0% (291)	52.8% (297)	**0.041**	**1.290 (1.011-1.647)**	70.9% (73)	**0.025**	**1.689 (1.065-2.679)**
Liver disease	29.6% (146)	19.9% (112)	**0.000**	**1.694 (1.276-2.249)**	42.7% (44)	**0.009**	**1.772 (1.146-2,74)**
Cancer/ immunological disease	48.1% (237)	36.1% (203)	**0.000**	**1.642 (1.283-2.101)**	53.4% (55)	0.325	1.238 (0.809-1.894)
Neurological disease	7.3% (36)	13.0% (73)	**0.003**	**0,529 (0,348-0,804)**	8.7% (9)	0.616	1.215 (0.566-2.608)
Charlson comorbidity index, median (IQR)	7 (4–9)	7 (4–9)	0.907	n.a.	7 (5–9)	0.377	n.a.
Length of stay as Median (IQR)							
LOS total (days)	42 (23–78)	32 (16–61)	**0.000**	n.a.	54 (36–85)	0.010	n.a.
LOS before BSI (days)	19 (9–34)	10 (2–25)	**0.000**	n.a.	27 (15–39)	**0.003**	n.a.
LOS after BSI (days)	18 (8–41)	16 (8–33)	0.308	n.a.	23 (8–45)	0.183	n.a.
LOS normal ward	15 (1–36)	11 (1–25)	**0.010**	n.a.	17 (0–44)	0.368	n.a.
LOS ICU	18 (1–49)	10 (1–40)	0.016	n.a.	24 (5–57)	0.050	n.a.
Hospital costs as median (IQR)							
Total hospital costs	51,365 (22,535-119,789)	31,122 (11,829-74,344)	**0.000**	n.a.	80,465 (47,887–157,447)	**0.000**	n.a.
Daily costs	1,237 (729–1,812)	1,014 (614–1,466)	**0.000**	n.a.	1,484 (1,095–2,186)	**0.000**	n.a.
Medical staff	7,600 (3169-16,468)	5,344 (1841-12,213)	**0.000**	n.a.	10,390 (6,364–20,983)	**0.002**	n.a.
Nursing staff	11,499 (4,476-26,237)	7,141 (2,610-21,591)	**0.000**	n.a.	16,661 (8,029–32,905)	**0.003**	n.a.
Assistant medical technicians	2,630 (1,028-5694)	1,906 (646–4,325)	**0.000**	n.a.	3,665 (1,397–6,795)	0.082	n.a.
Pharmacy	6,924 (2,200-20,922)	2,742 (636–7,865)	**0.000**	n.a.	17,145 (8,087–36,779)	**0.000**	n.a.
Expenses for implants/transplants	0 (0–412)	0 (0–170)	**0.005**	n.a.	20 (0–605)	0.767	n.a.
Medical supply	6,858 (2,820-15,057)	3,926 (− 1,338-9,782)	**0.000**	n.a.	9,878 (5,104–18,957)	**0.002**	n.a.
Medical infrastructure	1,893 (939–4,128)	1,473 (547–3,271)	**0.000**	n.a.	2,629 (1,611–5,219)	**0.001**	n.a.
Non-medical infrastructure	8,975 (4,215-18,206)	6,176 (2,576-14,049)	**0.000**	n.a.	12,244 (6,763–20,002)	**0.003**	n.a.

Categorical variables displayed as percentage and number; continuous variable displayed as median and interquartile range. *P-value, categorical variables tested with Chi-square test, continuous variable tested with Wilcoxon rank sum test. *BSI* = blood stream infection, *IQR* = interquartile range, *OR* = odds ratio, *CI95* = 95% confidence interval. *VRE* = vancomycin-resistant enterococcus, *VSE* = vancomycin-susceptible enterococcus. *N.a*. = not applicable

Bold entries represent statistically significant factors

**Fig. 2:**
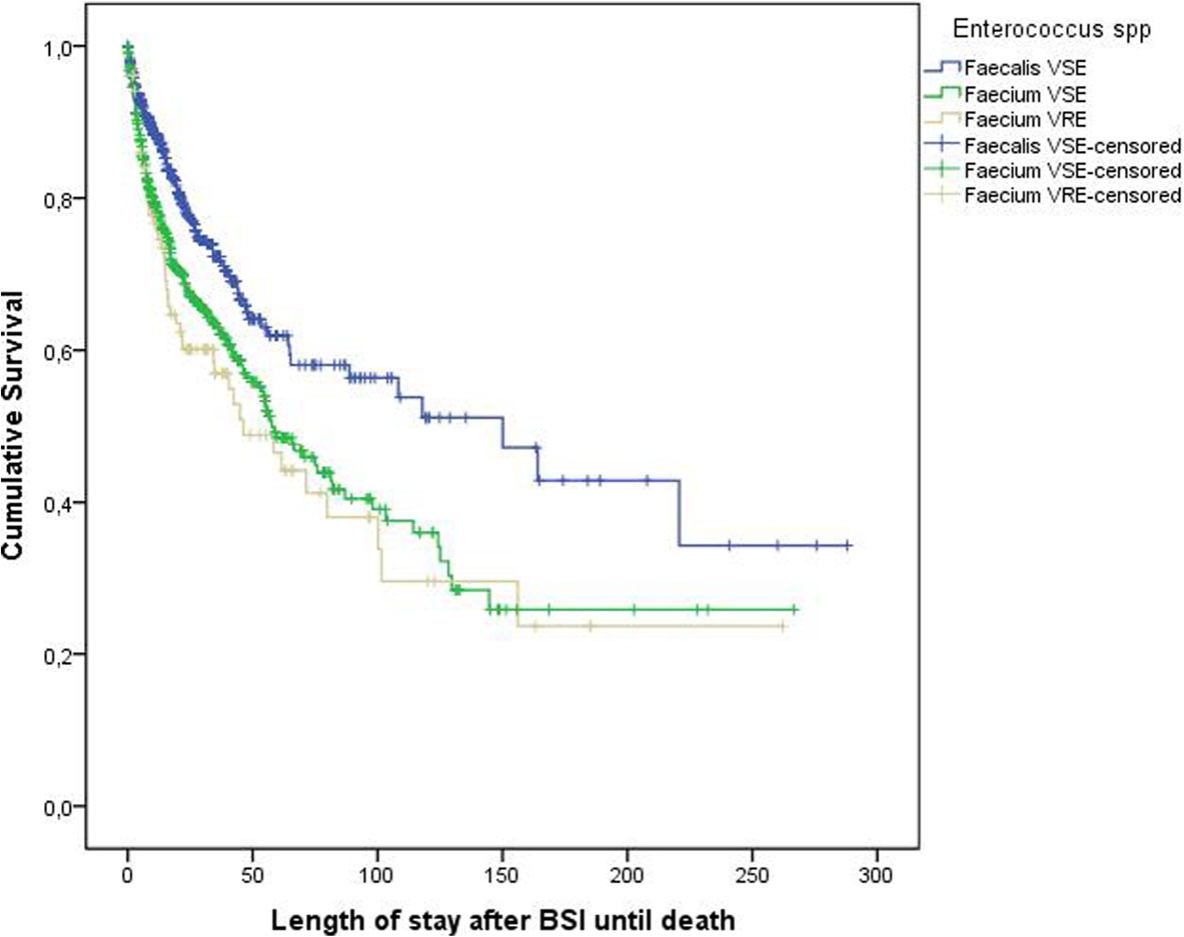
Kaplan Meier survival curve of patients with enterococcal blood stream infection (BSI). VSE, vancomycin-resistant enterococcus. VRE, vancomycin-susceptible enterococcus. Censored = left the hospital alive

In multivariable analyses on LOS after BSI onset among vancomycin-susceptible enterococci cases, some chronic diseases and *E. faecium* statistically significant increased LOS as compared to *E. faecalis* (see Table 2). Vancomycin-resistance was not found to additionally increase LOS among *E. faecium* cases (see Table 2 and Fig. 2). Regarding in-hospital death, patients with *E. faecium*-BSI had a higher chance for death as compared to *E. faecalis*-cases when only vancomycin-susceptible cases were considered (see Table 3 and 4). Among *E. faecium* cases, vancomycin-resistant was not found to be an additional risk factor for death (Table 4).

**Table 2:** Multivariable linear regression on length of stay of surviving patients after BSI onset

Parameter		VS-*E. faecium* vs. VS-*E. faecalis*					VS-*E. faecium* vs. VR-*E. faecium*				
		ME	Sig.	CI 95 (lower-upper)			ME	Sig.	CI 95 (lower-upper)		
Renal Disease		1.588	0000	1.891	–	3.868	1516	0.001	1.472	–	4.201
Age (years)		0.659	0000	0.969	–	0.987	Not significant				
Lung Disease		1.457	0000	1.800	–	4.342	1333	0.018	1.151	–	4.409
Gastrointestinal Disease		1.323	0001	1.959	–	11.025	Not significant				
Liver Disease		1.270	0.003	1.256	–	3.100	1370	0.009	1.226	–	4.155
Vascular Disease		1.216	0.016	1.104	–	2.622	Not significant				
Enterococcus species	*E. faecium*	1.258	0.004	1.170	–	2.324	Not applicable				
	*E. faecalis*	Reference = 1									

*BSI* = bloodstream infection, *CI95* = 95% confidence interval, *ME* multiplicative effect

**Table 3:** Univariate analysis on risk factors for in-hospital death

Parameter	Discharge alive (*n* = 781)	In-hospital death (*n* = 379)	*P-value	OR (CI 95)
Vancomycin resistance	6.7% (52)	13.7% (52)	**< 0.001**	**2.273 (1.512-3.417)**
*E. faecium*	44.7% (349)	65.2% (247)	**< 0.001**	**2.311 (1.792-2.979)**
E. faecalis	55.3% (432)	34.8% (132)		**1 = Reference**
Male	61.2% (478)	62.0% (235)	0.792	1.037 (0.805-1.334)
Age (years), median (IQR)	64.0 (51–73)	66.0 (56–74)	**0.011**	n.a.
Heart disease	25.1% (196)	35.4% (134)	**< 0.001**	**1.641 (1.258-2.14)**
Vascular disease	19.6% (153)	27.7% (105)	**0.002**	**1,57 (1,18-2.091)**
Lung disease	19.6% (153)	26.1% (99)	**0.011**	**1.461 (1.093-1.952)**
Rheumatic disease	2.6% (20)	4.7% (18)	0.050	1.895 (0,99-3.626)
Gastrointestinal disease	4.1% (32)	5.8% (22)	0.195	1,44 (0,825-2.515)
Diabetes	26.8% (209)	27.4% (104)	0.807	1.033 (0,784-1.361)
Renal disease	46.9% (366)	78.1% (296)	**< 0.001**	**4.055 (3.061-5.371)**
Liver disease	18.2% (142)	42.2% (160)	**< 0.001**	**3.283 (2.498-4.314)**
Cancer/Immunological disease	41.4% (323)	45.4% (172)	0.194	1.176 (0,918-1.506)
Neurological disease	10.8% (84)	9.0% (34)	0.346	0,817 (0.537-1.241)

Categorical variables displayed as percentage and number; continuous variable displayed as median and interquartile range. *P-value, categorical variables tested with Chi-square test, continuous variable tested with Wilcoxon rank sum test. *BSI* = blood stream infection, *IQR* = interquartile range, *OR* = odds ratio, *CI95* = 95% confidence interval. *VRE* = vancomycin-resistant enterococcus, *VSE* = vancomycin-susceptible enterococcus. *N.a.* = not applicable

Bold entries represent statistically significant factors

**Table 4:** Results of multivariable binary logistic regression of risk factors for in-hospital death after enterococcal bloodstream infection

Parameter		VS-*E. faecium* vs. VS-*E. faecalis*					VS-*E. faecium* vs. VR-*E. faecium*				
		OR	*P*-value	CI95 (lower-upper)			OR	*P*-value	CI95 (lower-upper)		
Age		1015	0,002	1005	–	1025	1016	0,010	1004	–	1029
Vascular disease		1407	0,042	1012	–	1956	Not significant				
Renal disease		3120	0,000	2274	–	4280	4005	0,000	2708	–	5922
Liver disease		2909	0,000	2109	–	4011	2390	0,000	1618	–	3529
Enterococcus species	VS-*E. faecium*	2023	0,000	1519	–	2695	Not applicable				
	VS-*E. faecalis*	Reference = 1					Not applicable				
Vancomycin-resistant		Not applicable					1.283	0.300	0.801	–	2.057
Vancomycin-susceptible		Not applicable					Reference = 1				

*OR* = odds ratio, *CI95* = 95% confidence interval

Regarding economic aspects, almost all hospital costs were significantly higher in the VREm BSI cohort compared to the VSEm BSI and the VSEf cohort.

## Discussion

During the last 15 years (since 2003), 4 meta-analyses on mortality- and financial burden of VRE infections [19, 22–24], and few recent studies on costs associated with VRE infections (not included in the meta-analyses) were published [13, 15, 32]. The studies demonstrate that vancomycin resistance is associated with overall worsened outcome (mortality, length of stay and hospital costs). However, although former studies indicated that *E. faecium* isolates might be more virulent than *E. faecalis* isolates irrespective of vancomycin resistance [2, 8, 9, 26], many of the above mentioned studies did not adjust for enterococcal subspecies. As Kaye et al. showed in 2004, this could lead to an overestimation of the outcome effects attributable to vancomycin resistance [9]. Some of the authors discussed this issue in their articles, arguing that meta-analyses cannot improve the quality of data published [22, 24].

In our analyses, in-hospital mortality and length of stay after BSI onset were independently associated with underlying diseases, age and *E. faecium* but not with vancomycin resistance.

Regarding mortality vancomycin resistance was associated with increased in-hospital mortality in the univariate analysis. This result is in agreement with recent studies including three meta-analyses [19, 22, 24]. However, after adjusting for underlying diseases, age, and species (*E. faecium* vs. *E. faecalis*), in-hospital mortality was no longer associated with VRE. There are several possible explanations for these differences with other studies.

Systemic enterococci infections mainly occur in patients with severe underlying diseases and comorbidities [33, 34] which also applies to our study cohort. Patients had a very high Charlson comorbidity score with a median of 7. In this regard, there was no significant difference between the cases infected with *E. faecalis*, *E. faecium* or vancomycin-resistant strains. Furthermore, infections caused by antimicrobial-resistant bacteria are often associated with increased morbidity and mortality [19]. These differences are explained by the delay in or even complete lack of an effective antibiotic treatment despite the availability of effective drugs [1, 24]. Prematunge et al. adjusted for appropriate antimicrobial therapy but found that the differences remained [24]. Outcome differences resulting from varying pathogenicity of enterococci species is an alternative explanation [35, 36]. In the multivariable analysis we found only small differences for in-hospital mortality resulting from vancomycin resistance, differences which are insufficient to explain the univariate results. However, we observed significantly increased mortality associated with vancomycin-susceptible *E. faecium* compared to vancomycin-susceptible *E. faecalis*.

Possibly because *E. faecium* is the most common type of VRE worldwide and most *E. faecalis* isolates from infections are vancomycin-susceptible, it might be a limitation to most existing studies that this confounder is not considered [10–16, 18–24, 32]. Prematunge et al. already pointed out in their 2016 meta-analysis, that it is possible that many observations on VRE-associated outcome are based on differences in the infection-causing species rather than on vancomycin resistance [24].

Attempts to assess the pathogenicity of enterococci go far back in time. In some of the previous studies, the pathogenic potential of commensal *E. faecium* was determined, whereas most of nowadays HAIs are caused by isolates of a different *E. faecium* lineage [37, 38]. These so-called hospital-associated strain types (formerly known as clonal complex CC17) differ from commensal human and animal isolates by a distinct core and accessory genome content. Ampicillin resistance is a phenotypic marker of these hospital-associated strain types [39–41]. It has been shown that AMP-R *E. faecium* isolates causing healthcare-associated infections are in fact more pathogenic than commensal variants [42, 43]. In a supplementary analysis (Additional file 1: Tables S1 and S2) we found that 95% of our *E. faecium* isolates were ampicillin-resistant (AMP-R), in contrast to only 1% of the *E. faecalis* isolates. Due to the uneven distribution of ampicillin resistance over the two pathogens we were not able to assess potential virulence differences between commensal and hospital-associated isolates. However, our results are supportive to previous population-based and molecular analyses of *E. faecium* from hospital-acquired infections.

Several reports have linked VRE infections with increased costs [13, 15, 19, 32]. In the univariate analyses, our data showed similar results as almost all costs were significantly higher in VREm-BSI patients than in patients with VSEm-BSI. Butler et al. reported that pharmacy costs are the second most relevant driver of increased costs, making up to 18% of the total amount [21]. In our cohort, the percentage of pharmaceutical costs for patients with VREm made up 21% of the total hospital costs while they made up only 9% of the costs of patents with BSI caused by VSEm (< 0,001). As we were not able to attribute costs to particular agents, these differences could be due to higher antibiotics costs or to differences in underlying conditions treated. For this reason, we analyzed the length of stay before and after onset of BSI. Our data showed increased lengths of stay overall and before onset of VREm-BSI, but not after. Interestingly, all BSIs were classified as HAIs as none occurred prior to day 3 after hospital admission. On average, the BSI episodes occurred on day 16 of hospitalization. Moreover, the same phenomenon was observed in the analysis of the infection-attributable LOS stratified by the two clinically relevant Enterococcus species. Cases with vancomycin susceptible *E. faecium* BSI were in-hospital longer before onset of infection than vancomycin susceptible *E. faecalis* cases. No difference in LOS was observed after onset of infection.

This study has several limitations. The cases were identified retrospectively through a microbiological database and 6.6% lacked sufficient data for this analysis. We did not have data on the course or severity of infection and the antibiotic treatment performed. We did not have separate data on true costs before and after the bloodstream infection. We did not perform molecular analyses on the enterococci isolates to assess their potential virulence traits.

## Conclusion

In our study, vancomycin resistance in patients with *Enterococcus faecium* bloodstream infection was associated with increased total costs, length of stay before onset of infection, but not with infection-attributable LOS or in-hospital mortality. We observed that vancomycin-susceptible *E. faecium* infections were more strongly associated with increased LOS and mortality than vancomycin susceptible *E. faecalis* infections and might indicate higher virulence of *E. faecium* as compared to *E. faecalis.* In order to avoid overestimation of VRE-attributable effects, in addition to vancomycin-resistance species should be taken into consideration in future studies assessing the burden of VRE infections.

## Additional file

Additional file 1: **Table S1.** Susceptibility towards Ampicillin among cases. **Table S2.** Susceptibility towards Ampicillin among Isolate n=193 missing; AMP= Susceptibility to Ampicillin. R=Resistant. S=Susceptible. RR= relative risk. CI95=95% confidence interval. (DOCX 34 kb)

## Abbreviations

95% CI: 95% confidence interval
BSI: blood stream infection
CCI: Charlson comorbidity index
HAI: hospital acquired infection
HR: Hazard ratios
IQR: interquartile range
LOS: length of stay
ME: multiplicative effect
VRE: vancomycin-resistant enterococcus
VREm: vancomycin resistant *Enterococcus faecium*
VSE: vancomycin-susceptible enterococcus
VSEf: vancomycin susceptible *E. faecalis*
VSEm: vancomycin susceptible *Enterococcus faecium*
