# Supplementary material for: The importance of adjusting for enterococcus species when assessing the burden of vancomycin resistance: a cohort study including over 1000 cases of enterococcal bloodstream infections
Source: Antimicrob Resist Infect Control. 2018 Nov 14;7:133. doi: 10.1186/s13756-018-0419-9 (PMC6234683; doi:10.1186/s13756-018-0419-9)
Supplement: Supplementary file 1 — Table S1. Susceptibility towards Ampicillin among cases. Table S2. Susceptibility towards Ampicillin among Isolate n=193 missing; AMP= Susceptibility to Ampicillin. R=Resistant. S=Susceptible. RR= relative risk. CI95=95% confidence interval. (DOCX 34 kb) [file 13756_2018_419_MOESM1_ESM.docx]

Table S1

|  |  | In-hospital death | |  |  |  |  |
| --- | --- | --- | --- | --- | --- | --- | --- |
|  |  | 1 | 0 |  | P-value | RR | CI95 |
| AMP | R | 40% (n=195) | 60% (n=288) | 100% (n=483) | <0.001 | 1.760 | 1.447-2.142 |
|  | S | 23% (n=111) | 77% (n=373) | 100% (n=484) |  |  |  |
|  |  | n=306 | n=661 | n=967 |  |  |  |
|  |  |  |  |  |  |  |  |
| n=193 missing | | | | | | | |

Table S2

|  |  | AMP | |  |  |  |  |
| --- | --- | --- | --- | --- | --- | --- | --- |
|  |  | S | R |  | P-value | RR | CI95 |
|  | *E. faecium* | 5% (n=26) | 95% (n=478) | 100% (n=504) | <0.001 | 0.052 | 0.036-0.076 |
|  | *E. faecalis* | 99% (n=458) | 1% (n=5) | 100% (n=463) |  |  |  |
|  |  | n=484 | n=483 | n=967 |  |  |  |
|  |  |  |  |  |  |  |  |
| n=193 missing | | | | | | | |
